# Supplementary figures and images for: Playing by the rules? Phenotypic adaptation to temperate environments in an American marsupial
Source: PeerJ. 2018 Mar 27;6:e4512. doi: 10.7717/peerj.4512 (PMC5877449; doi:10.7717/peerj.4512)

A

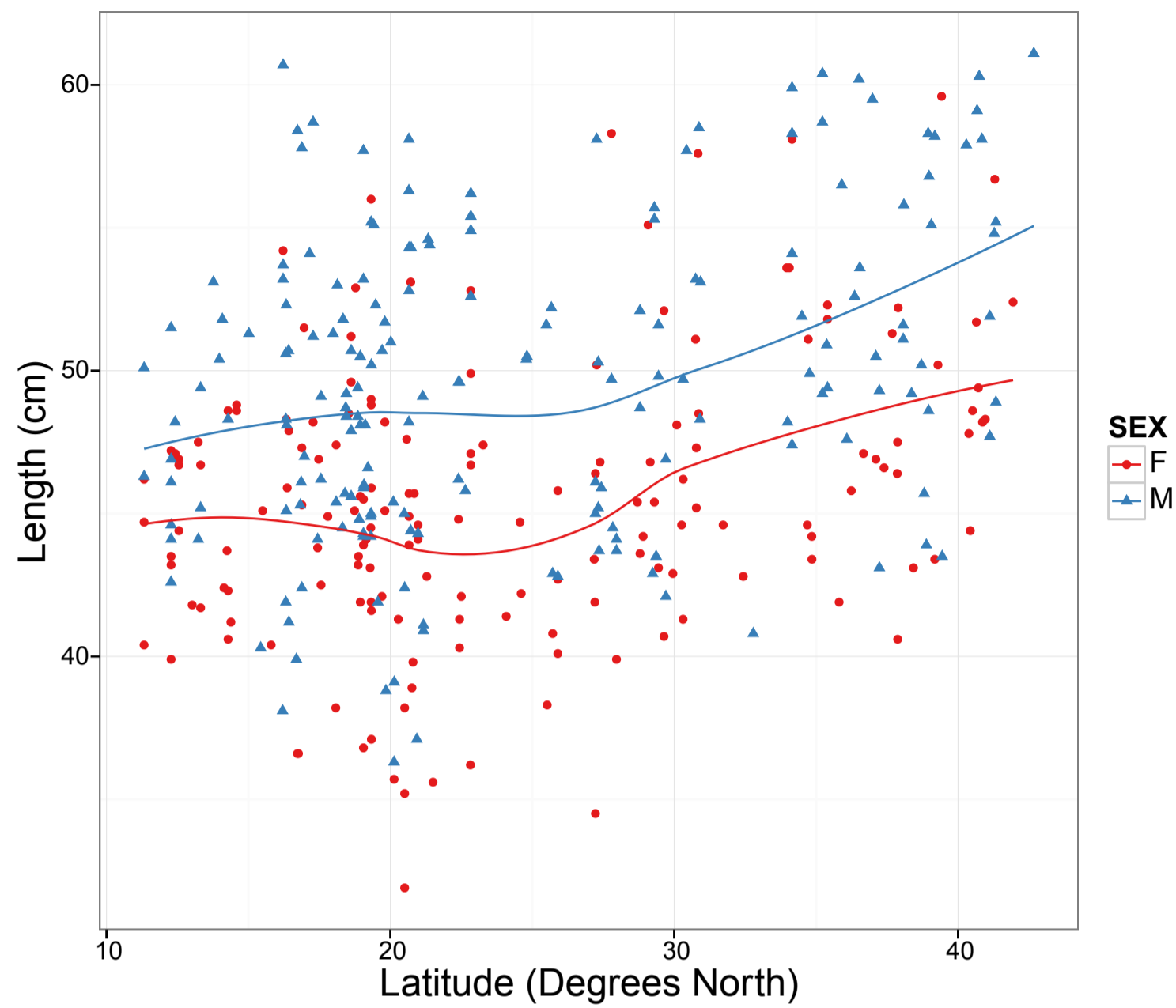

B

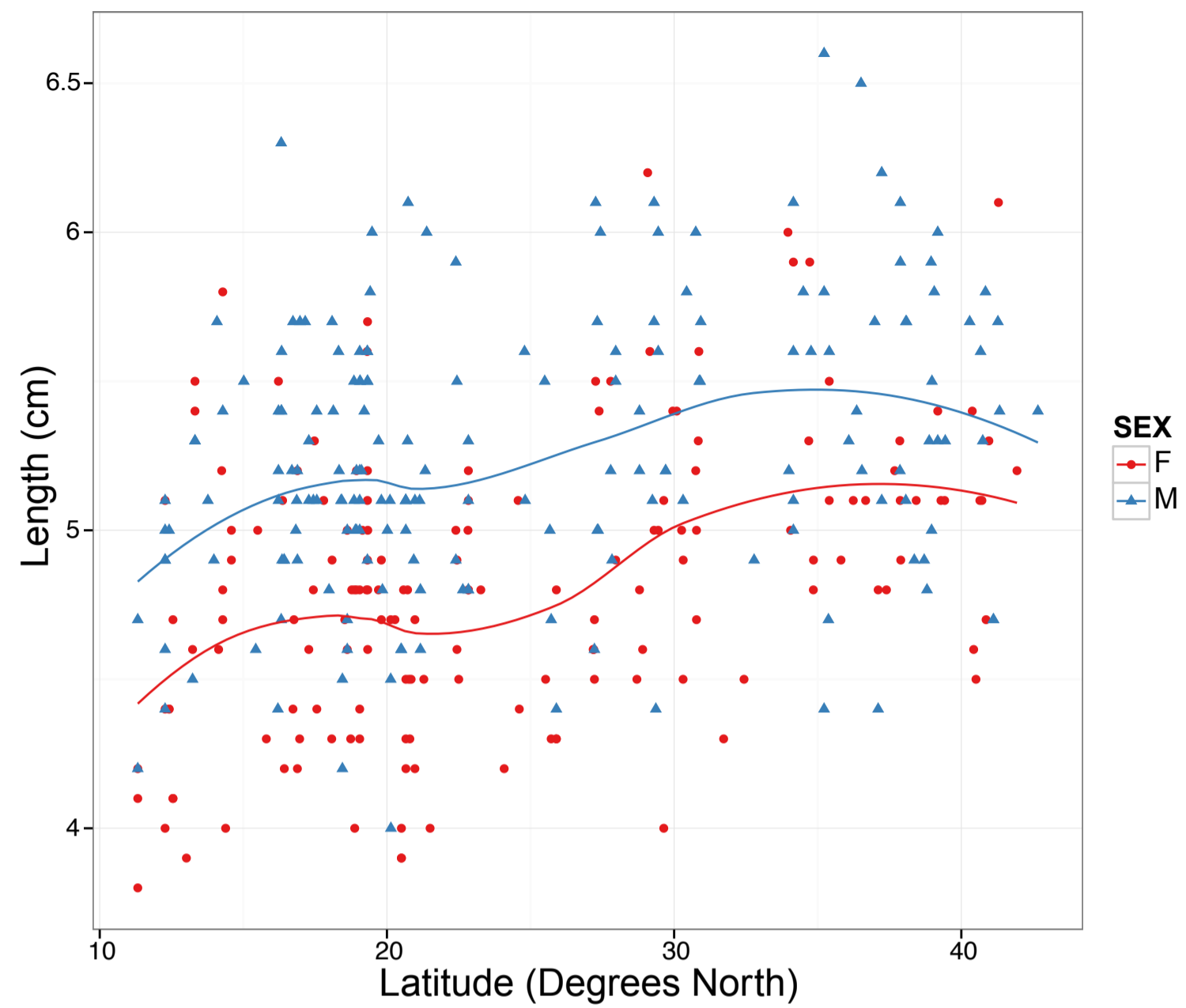

C

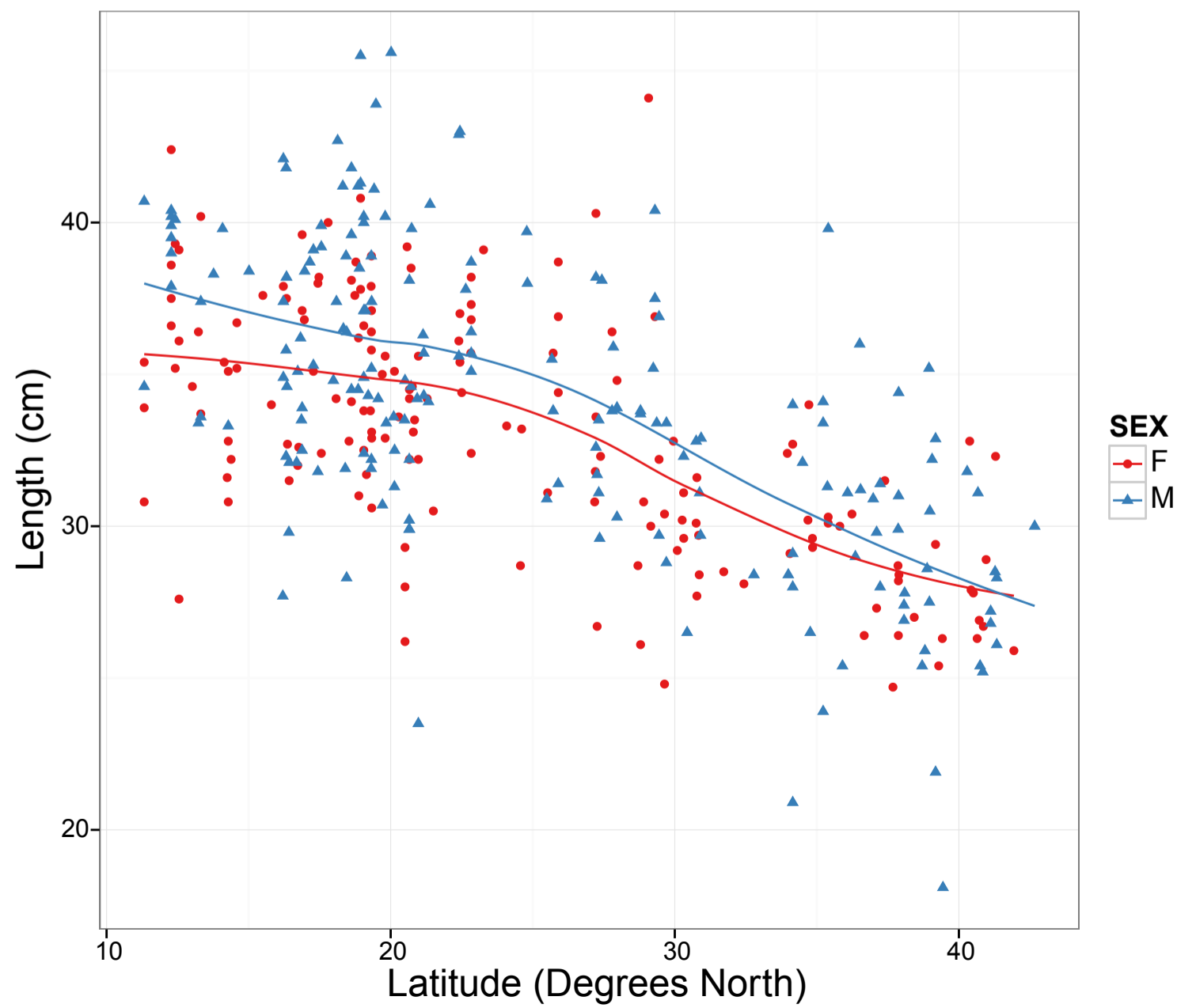

D

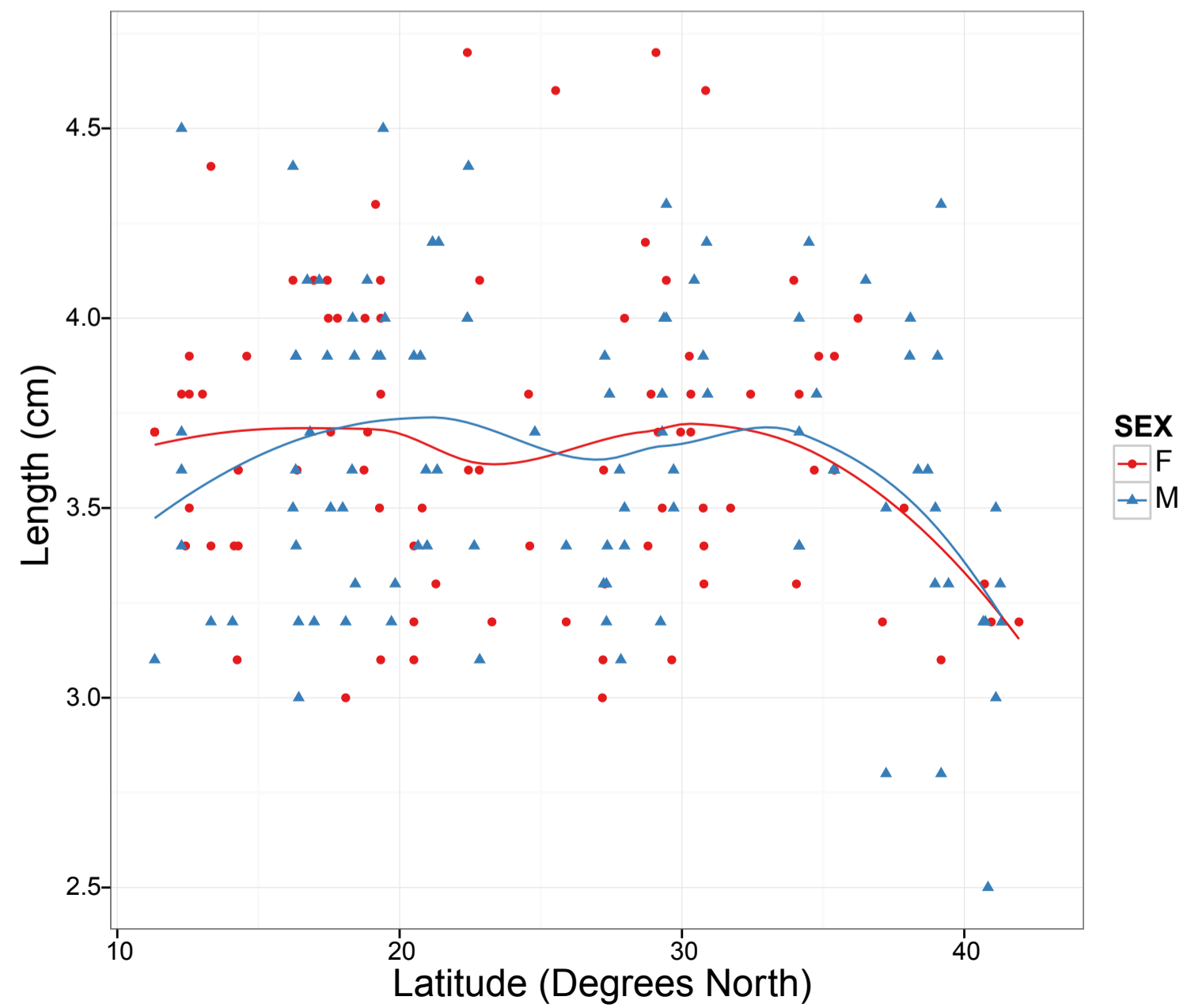

Supplement: Figure S1 — The non-linear loess function line is shown, indicating the trend of the relationship. (A) body length, (B) hindfoot length, (C) tail length, (D) ear length. F, females; M, males. [file peerj-06-4512-s002.pdf]
