# Supplementary material for: Playing by the rules? Phenotypic adaptation to temperate environments in an American marsupial
Source: PeerJ. 2018 Mar 27;6:e4512. doi: 10.7717/peerj.4512 (PMC5877449; doi:10.7717/peerj.4512)
Supplement: Table S1 — N measured, Number of specimens measured; N analyzed, Number of specimens included in the analyses; F, Females; M, Males. [file peerj-06-4512-s003.docx]

**Table S1.** Total number of male and female *Didelphis virginiana* specimens measured and analyzed for all dimension and pigmentation traits. N measured = Number of specimens measured, N analyzed = Number of specimens included in the analyses, F = Females, M = Males.

| Trait | N measured |  | N analyzed |
| --- | --- | --- | --- |
|  | F M Total |  | F M Total |
| Body length | 163 189 352 |  | 163 185 348 |
| Hindfoot length | 163 189 352 |  | 160 185 345 |
| Tail length | 163 189 352 |  | 162 186 348 |
| Ear length | 163 189 352 |  | 84 101 185 |
| Tail pigmentation | 159 186 345 |  | 157 183 340 |
| Ear pigmentation | 159 186 345 |  | 134 159 293 |
| Toe ventral pigmentation | 159 186 345 |  | 151 183 334 |
| Toe dorsal pigmentation | 159 186 345 |  | 155 183 338 |
| Rostrum lightness | 159 186 345 |  | 159 186 345 |
| Temporal lightness | 159 186 345 |  | 159 186 345 |
| Cheek lightness | 159 186 345 |  | 159 186 345 |
| Torso lightness | 159 186 345 |  | 159 186 345 |
