# Supplementary material for: Playing by the rules? Phenotypic adaptation to temperate environments in an American marsupial
Source: PeerJ. 2018 Mar 27;6:e4512. doi: 10.7717/peerj.4512 (PMC5877449; doi:10.7717/peerj.4512)
Supplement: Table S2 [file peerj-06-4512-s004.docx]

**Table S2**. List of all *Didelphis virginiana* museum specimens of used in our study. The specimen numbers were recorded as shown in the specimen tags. In collection, AMNH = American Museum of Natural History, CNMA = Colección Nacional de Mamíferos (UNAM, Mexico), MALH = Museo de Zoología “Alfonso L. Herrera” (UNAM. Mexico), MVZ = Museum of Vertebrate Zoology (UC-Berkeley), USNM = Smithsonian Museum of Natural History. In country, BEL = Belize, GUA = Guatemala, HON = Honduras, MEX = Mexico, NIC = Nicaragua, SLV =El Salvador, USA = United States of America. In sex, F = Females, M = Males. N/A = Not available.

| Collection | Specimen Number | Collecting year | Country | Latitude | Longitude | Sex |
| --- | --- | --- | --- | --- | --- | --- |
| AMNH | 42270 | 1917 | USA | 40.380755 | -84.864867 | F |
| AMNH | 135053 | 1942 | USA | 39.179033 | -96.597909 | M |
| AMNH | 142645 | 1943 | USA | 39.179033 | -96.597909 | M |
| AMNH | 142644 | 1943 | USA | 39.179033 | -96.597909 | F |
| AMNH | 121735 | 1935 | USA | 41.937622 | -85.011236 | F |
| AMNH | 9071/7378 | 1894 | USA | 40.670676 | -74.208794 | M |
| AMNH | 3596/2791 | 1891 | USA | 40.716771 | -74.011413 | F |
| AMNH | 16664 | 1901 | USA | 41.332225 | -73.987078 | M |
| AMNH | 16608 | 1900 | USA | 42.667253 | -74.319322 | M |
| AMNH | 16666 | 1901 | USA | 41.332225 | -73.987078 | M |
| AMNH | 180142 | 1959 | USA | 41.11812 | -73.798402 | M |
| AMNH | 180141 | 1959 | USA | 41.11812 | -73.798402 | M |
| AMNH | 1233 | 1887 | USA | 40.958687 | -72.992222 | F |
| AMNH | 146632 | 1950 | USA | 41.292679 | -73.669186 | F |
| AMNH | 129388 | 1938 | USA | 41.273651 | -73.77428 | M |
| AMNH | 16594 | 1899 | USA | 35.221196 | -75.686696 | M |
| AMNH | 16595 | 1899 | USA | 35.221196 | -75.686696 | M |
| AMNH | 16596 | 1899 | USA | 35.221196 | -75.686696 | M |
| AMNH | 147741 | 1951 | USA | 35.401311 | -78.81566 | M |
| AMNH | 147743 | 1951 | USA | 35.401311 | -78.81566 | F |
| AMNH | 147744 | 1951 | USA | 35.401311 | -78.81566 | F |
| AMNH | 149377 | 1952 | USA | 35.809671 | -83.637353 | F |
| AMNH | 8653 | 1895 | USA | 29.446438 | -98.472335 | M |
| AMNH | 8651 | 1895 | USA | 29.446438 | -98.472335 | M |
| AMNH | 14827 | 1897 | USA | 29.446438 | -98.472335 | F |
| AMNH | 126141 | 1936 | HON | 14.284046 | -87.257673 | F |
| AMNH | 126189 | 1937 | HON | 14.284046 | -87.257673 | F |
| AMNH | 146585 | 1949 | BEL | 18.400705 | -88.389935 | M |
| AMNH | 128477 | 1937 | HON | 14.284046 | -87.257673 | M |
| AMNH | 128476 | 1937 | HON | 14.284046 | -87.257673 | F |
| AMNH | 123286 | 1935 | HON | 14.08223 | -87.214262 | M |
| AMNH | 126193 | 1936 | HON | 14.246064 | -87.955458 | F |
| AMNH | 172153 | 1956 | MEX | 17.155095 | -92.900016 | M |
| AMNH | 172160 | 1956 | MEX | 16.728409 | -92.627128 | M |
| AMNH | 126191 | 1936 | HON | 14.139676 | -87.908227 | F |
| AMNH | 128974 | 1937 | HON | 14.585282 | -88.58484 | F |
| AMNH | 128976 | 1937 | HON | 14.585282 | -88.58484 | F |
| AMNH | 25181 | 1905 | MEX | 20.654136 | -102.329135 | M |
| AMNH | 25182 | 1905 | MEX | 20.654136 | -102.329135 | M |
| AMNH | 25183 | 1905 | MEX | 20.654136 | -102.329135 | M |
| AMNH | 16625 | 1900 | MEX | 20.659814 | -103.346833 | F |
| AMNH | 16626 | 1900 | MEX | 20.659814 | -103.346833 | F |
| AMNH | 16629 | 1900 | MEX | 20.659814 | -103.346833 | F |
| AMNH | 26017 | 1905 | MEX | 19.709972 | -103.470899 | M |
| AMNH | 25184 | 1905 | MEX | 20.657412 | -104.509528 | M |
| AMNH | 25188 | 1905 | MEX | 20.657412 | -104.509528 | M |
| AMNH | 25852 | 1905 | MEX | 20.577607 | -104.432894 | F |
| AMNH | 145179 | 1947 | MEX | 16.22253 | -94.855967 | M |
| AMNH | 145951 | 1948 | MEX | 16.318396 | -94.753993 | M |
| AMNH | 145180 | 1947 | MEX | 16.22253 | -94.855967 | M |
| AMNH | 145181 | 1947 | MEX | 16.22253 | -94.855967 | F |
| AMNH | 145629 | 1948 | MEX | 16.22253 | -94.855967 | M |
| AMNH | 145175 | 1947 | MEX | 16.426591 | -95.410184 | F |
| AMNH | 145176 | 1947 | MEX | 16.426591 | -95.410184 | M |
| AMNH | 145956 | 1949 | MEX | 16.332628 | -95.22265 | M |
| AMNH | 145634 | 1948 | MEX | 16.417467 | -95.332708 | M |
| AMNH | 145955 | 1949 | MEX | 16.332762 | -95.227662 | M |
| AMNH | 145958 | 1949 | MEX | 16.332628 | -95.22265 | F |
| AMNH | 24717 | 1904 | MEX | 22.835248 | -105.787437 | F |
| AMNH | 24718 | 1904 | MEX | 22.835248 | -105.787437 | M |
| AMNH | 24720 | 1904 | MEX | 22.835248 | -105.787437 | F |
| AMNH | 131205 | 1938 | USA | 30.778987 | -84.95078 | F |
| AMNH | 131206 | 1938 | USA | 30.778987 | -84.95078 | F |
| AMNH | 91144 | 1929 | USA | 30.86181 | -82.258406 | F |
| AMNH | 182029 | 1951 | USA | 27.791546 | -82.641085 | F |
| AMNH | 100088 | 1931 | USA | 30.840257 | -83.96581 | F |
| AMNH | 93125 | 1930 | USA | 30.873395 | -83.84229 | M |
| AMNH | 24722 | 1904 | MEX | 22.835248 | -105.787437 | M |
| AMNH | 24725 | 1904 | MEX | 22.835248 | -105.787437 | F |
| AMNH | 24726 | 1904 | MEX | 22.835248 | -105.787437 | M |
| AMNH | 24727 | 1904 | MEX | 22.835248 | -105.787437 | M |
| AMNH | 146770 | 1949 | MEX | 24.082936 | -99.110274 | F |
| AMNH | 172164 | 1956 | MEX | 18.775154 | -95.759372 | F |
| AMNH | 172157 | 1956 | MEX | 17.438123 | -95.023441 | M |
| AMNH | 172159 | 1956 | MEX | 17.438123 | -95.023441 | F |
| AMNH | 30524 | 1905 | MEX | 20.974654 | -89.613131 | M |
| AMNH | 1899/1164 | 1889 | USA | 29.63898 | -82.32214 | F |
| AMNH | 1900/1165 | 1889 | USA | 29.63898 | -82.32214 | F |
| AMNH | 163976 | 1951 | USA | 28.795785 | -82.589785 | F |
| AMNH | 243428 | 1941 | USA | 27.181218 | -81.35071 | F |
| AMNH | 243429 | 1941 | USA | 27.267314 | -81.364121 | F |
| AMNH | 243430 | 1941 | USA | 27.267314 | -81.364121 | M |
| AMNH | 131201 | 1938 | USA | 30.315848 | -84.134981 | F |
| AMNH | 131204 | 1938 | USA | 30.434785 | -84.271829 | M |
| CNMA | 34858 | 1992 | MEX | 19.323422 | -99.185176 | M |
| CNMA | 43374 | 2006 | MEX | 19.3125 | -99.188056 | F |
| CNMA | 43372 | 2005 | MEX | 19.3125 | -99.188056 | M |
| CNMA | 4254 | 1989 | MEX | 19.332633 | -99.185517 | F |
| CNMA | 33417 | 1990 | MEX | 19.332633 | -99.185517 | F |
| CNMA | 23070 | 1998 | MEX | 19.317839 | -99.184141 | M |
| CNMA | 45114 | 2008 | MEX | 19.320667 | -99.177722 | M |
| CNMA | 45113 | 2009 | MEX | 19.322528 | -99.194722 | F |
| CNMA | 45116 | 2008 | MEX | 19.325278 | -99.189167 | F |
| CNMA | 4159 | 1986 | MEX | 20.8421917 | -100.7442474 | F |
| CNMA | 21866 | 1985 | MEX | 21.140256 | -100.066275 | M |
| CNMA | 3522 | 1979 | MEX | 16.6900005 | -99.6255569 | M |
| CNMA | 15637 | 1974 | MEX | 16.8272228 | -99.8047256 | M |
| CNMA | 45117 | 2009 | MEX | 16.972472 | -100.014722 | M |
| CNMA | 11687 | 1970 | MEX | 18.3152771 | -99.9300003 | M |
| CNMA | 42921 | 2005 | MEX | 19.561389 | -105.083333 | M |
| CNMA | 3788 | 1982 | MEX | 19.481741 | -98.823527 | M |
| CNMA | 9758 | 1967 | MEX | 19.0705547 | -99.3308334 | M |
| CNMA | 26454 | 1985 | MEX | 18.8677769 | -99.4402771 | F |
| CNMA | 26460 | 1985 | MEX | 18.8516674 | -99.4122238 | M |
| CNMA | 1193 | 1952 | MEX | 25.673344 | -100.341523 | M |
| CNMA | 45141 | 2007 | MEX | 18.133611 | -97.825278 | M |
| CNMA | 2475 | 1956 | MEX | 16.3669453 | -94.1944427 | F |
| CNMA | 3790 | 1984 | MEX | 20.106589 | -97.356429 | M |
| CNMA | 21991 | 1985 | MEX | 22.4381561 | -99.3056946 | F |
| CNMA | 21989 | 1985 | MEX | 22.4381561 | -99.3056946 | F |
| CNMA | 21990 | 1985 | MEX | 22.4381561 | -99.3056946 | M |
| CNMA | 45120 | 2008 | MEX | 22.821278 | -105.787556 | F |
| CNMA | 26116 | 1975 | MEX | 24.6095352 | -98.7221832 | F |
| CNMA | 45124 | 2008 | MEX | 18.619164 | -95.661222 | M |
| CNMA | 38011 | 1995 | MEX | 20.0162354 | -97.1416092 | M |
| CNMA | 45123 | 2008 | MEX | 18.619164 | -95.661222 | M |
| CNMA | 45125 | 2008 | MEX | 18.619164 | -95.661222 | F |
| CNMA | 45126 | 2008 | MEX | 18.619164 | -95.661222 | F |
| CNMA | 45127 | 2008 | MEX | 18.619164 | -95.661222 | M |
| MALH | 3466 | 1984 | MEX | 21.332467 | -99.436397 | M |
| MALH | 3465 | 1985 | MEX | 19.321558 | -99.190207 | F |
| MALH | 3472 | 1984 | MEX | 18.738847 | -99.443999 | F |
| MALH | 3468 | 1982 | MEX | 19.321558 | -99.190207 | F |
| MALH | 1397 | 1983 | MEX | 21.284622 | -99.470073 | F |
| MALH | 3475 | 1984 | MEX | 18.901625 | -95.955748 | M |
| MALH | 3473 | 1984 | MEX | 19.330151 | -96.628094 | M |
| MALH | 45 | 1975 | MEX | 18.335131 | -94.750667 | M |
| MALH | 3476 | 1984 | MEX | 19.330151 | -96.628094 | F |
| MALH | 925 | 1983 | MEX | 17.474755 | -100.173364 | F |
| MALH | 931 | 1985 | MEX | 17.555115 | -99.685619 | M |
| MVZ | 100067 | 1943 | MEX | 19.14769116 | -101.4421185 | F |
| MVZ | 100074 | 1943 | MEX | 19.20953492 | -101.45667 | M |
| MVZ | 91164 | 1940 | MEX | 25.52559527 | -100.30833 | F |
| MVZ | 85261 | 1939 | MEX | 27.225 | -109.25889 | M |
| MVZ | 85262 | 1939 | MEX | 27.225 | -109.25889 | F |
| MVZ | 121179 | 1955 | MEX | 18.53222222 | -95.30722222 | F |
| MVZ | 85264 | 1939 | MEX | 27.225 | -109.25889 | M |
| MVZ | 85263 | 1939 | MEX | 27.225 | -109.25889 | F |
| MVZ | 81582 | 1937 | USA | 30.089 | -94.144 | F |
| MVZ | 33460 | 1923 | USA | 37.8724816 | -122.2453117 | F |
| MVZ | 47148 | 1931 | USA | 37.8634074 | -122.2148874 | M |
| MVZ | 51974 | 1932 | USA | 37.8761067 | -122.2546404 | M |
| MVZ | 97345 | 1941 | USA | 37.8788042 | -122.265829 | M |
| MVZ | 98145 | 1942 | USA | 37.89053 | -122.27374 | F |
| MVZ | 126168 | 1960 | USA | 37.85754 | -122.2316 | F |
| MVZ | 220246 | 2006 | USA | 37.870098 | 122.2449 | F |
| MVZ | 138994 | 1968 | USA | 39.4184056 | -122.1628702 | F |
| MVZ | 104560 | 1946 | USA | 36.9841 | -120.5341 | M |
| MVZ | 77023 | 1937 | USA | 34.056 | -117.182 | F |
| MVZ | 21990 | 1892 | USA | 38.9633 | -95.235 | M |
| MVZ | 106578 | 1946 | USA | 40.645 | -97.4503 | F |
| MVZ | 114854 | 1944 | USA | 40.5105 | -96.1637 | F |
| MVZ | 114842 | 1944 | USA | 40.8619 | -96.6701 | F |
| MVZ | 129731 | 1961 | USA | 36.0816 | -80.2506 | M |
| MVZ | 80746 | 1938 | USA | 35.90302 | -98.48426 | M |
| MVZ | 81408 | 1937 | USA | 39.4397 | -83.8367 | M |
| MVZ | 31840 | 1918 | USA | 38.8822 | -77.1714 | M |
| MVZ | 14704 | 1910 | USA | 37.104081 | -76.46406 | M |
| MVZ | 14705 | 1910 | USA | 37.104081 | -76.46406 | F |
| MVZ | 130275 | 1927 | SLV | 14.38333 | -89.13333 | F |
| MVZ | 98152 | 1942 | SLV | 13.76667 | -88.21667 | M |
| MVZ | 98155 | 1942 | SLV | 13.31667 | -88.06667 | M |
| MVZ | 130298 | 1925 | SLV | 13.31667 | -88.06667 | F |
| MVZ | 130299 | 1925 | SLV | 13.31667 | -88.06667 | F |
| MVZ | 130302 | 1925 | SLV | 13.31667 | -88.06667 | M |
| MVZ | 130310 | 1926 | SLV | 13.23333 | -88.36667 | M |
| MVZ | 130311 | 1926 | SLV | 13.23333 | -88.36667 | F |
| USNM | 76717 | 1895 | GUA | 15.801345 | -91.75282 | F |
| USNM | 275678 | 1947 | GUA | 13.975257 | -91.056542 | M |
| USNM | 19463 | 1891 | HON | 15.501307 | -88.028649 | F |
| USNM | 148748 | 1901 | HON | 15.432392 | -88.011173 | M |
| USNM | 181261 | 1913 | MEX | 18.449316 | -90.106533 | M |
| USNM | 181262 | 1913 | MEX | 18.449316 | -90.106533 | M |
| USNM | 76202 | 1895 | MEX | 16.763288 | -93.364336 | F |
| USNM | 76203 | 1895 | MEX | 16.859337 | -93.412168 | M |
| USNM | 76209 | 1895 | MEX | 16.728409 | -92.627128 | F |
| USNM | 76211 | 1895 | MEX | 17.280126 | -92.31423 | M |
| USNM | 76213 | 1895 | MEX | 17.280126 | -92.31423 | F |
| USNM | 76214 | 1895 | MEX | 17.280126 | -92.31423 | M |
| USNM | 76716 | 1895 | MEX | 16.204405 | -92.087984 | M |
| USNM | 78001 | 1896 | MEX | 15.015922 | -92.393241 | M |
| USNM | 133187 | 1904 | MEX | 16.347409 | -92.549229 | M |
| USNM | 32635 | 1892 | MEX | 19.052582 | -104.3199 | F |
| USNM | 32636 | 1892 | MEX | 19.052582 | -104.3199 | M |
| USNM | 32637 | 1892 | MEX | 19.052582 | -104.3199 | M |
| USNM | 32639 | 1892 | MEX | 19.052582 | -104.3199 | F |
| USNM | 32645 | 1892 | MEX | 19.052582 | -104.3199 | M |
| USNM | 32646 | 1892 | MEX | 19.052582 | -104.3199 | F |
| USNM | 33226 | 1892 | MEX | 19.052582 | -104.3199 | M |
| USNM | 33264 | 1892 | MEX | 18.940487 | -103.959676 | F |
| USNM | 33265 | 1892 | MEX | 18.940487 | -103.959676 | M |
| USNM | 33266 | 1892 | MEX | 18.940487 | -103.959676 | F |
| USNM | 33268 | 1892 | MEX | 18.940487 | -103.959676 | M |
| USNM | 96819 | 1899 | MEX | 24.812327 | -106.739015 | M |
| USNM | 70617 | 1895 | MEX | 16.883054 | -99.887943 | M |
| USNM | 70618 | 1895 | MEX | 16.883054 | -99.887943 | F |
| USNM | 70619 | 1895 | MEX | 16.883054 | -99.887943 | M |
| USNM | 70620 | 1895 | MEX | 16.883054 | -99.887943 | F |
| USNM | 126715 | 1903 | MEX | 18.078074 | -101.991479 | F |
| USNM | 26418 | 1893 | MEX | 20.131751 | -98.74074 | M |
| USNM | 26419 | 1893 | MEX | 20.131751 | -98.74074 | F |
| USNM | 26420 | 1893 | MEX | 20.13823 | -98.673559 | M |
| USNM | 81726 | 1896 | MEX | 20.489274 | -99.214157 | M |
| USNM | 33517 | 1892 | MEX | 19.704722 | -103.447259 | F |
| USNM | 34338 | 1892 | MEX | 20.718654 | -103.364894 | F |
| USNM | 34339 | 1892 | MEX | 20.718654 | -103.364894 | M |
| USNM | 88142 | 1892 | MEX | 20.76192 | -104.84995 | F |
| USNM | 51506 | 1893 | MEX | 19.115792 | -98.760162 | M |
| USNM | 20443 | 1892 | MEX | 20.27995 | -102.473747 | F |
| USNM | 35526 | 1892 | MEX | 19.806169 | -100.890701 | F |
| USNM | 35527 | 1892 | MEX | 19.806169 | -100.890701 | F |
| USNM | 35528 | 1892 | MEX | 19.806169 | -100.890701 | M |
| USNM | 126167 | 1903 | MEX | 19.057792 | -101.607015 | M |
| USNM | 51125 | 1893 | MEX | 18.878628 | -99.058899 | F |
| USNM | 25558 | 1891 | MEX | 25.71894 | -100.404588 | F |
| USNM | 25735 | 1891 | MEX | 25.71894 | -100.404588 | M |
| USNM | 88143 | 1897 | MEX | 21.503094 | -104.898364 | F |
| USNM | 91169 | 1897 | MEX | 22.501016 | -105.363347 | F |
| USNM | 512183 | 1976 | MEX | 20.797837 | -105.22071 | F |
| USNM | 523002 | 1977 | MEX | 21.166813 | -105.227379 | M |
| USNM | 523003 | 1977 | MEX | 21.166813 | -105.227379 | M |
| USNM | 553883 | 1981 | MEX | 21.384945 | -105.190793 | M |
| USNM | 65954 | 1894 | MEX | 18.09047 | -96.131845 | M |
| USNM | 65955 | 1894 | MEX | 18.09047 | -96.131845 | F |
| USNM | 69798 | 1894 | MEX | 17.798372 | -96.962691 | F |
| USNM | 73490 | 1895 | MEX | 16.318736 | -95.240935 | M |
| USNM | 73491 | 1895 | MEX | 16.961302 | -95.096382 | F |
| USNM | 73492 | 1892 | MEX | 16.318736 | -95.240935 | M |
| USNM | 55579 | 1893 | MEX | 19.287397 | -98.430622 | F |
| USNM | 92978 | 1898 | MEX | 20.736921 | -97.85191 | M |
| USNM | 96225 | 1898 | MEX | 27.202507 | -107.712006 | F |
| USNM | 96820 | 1899 | MEX | 24.790704 | -107.386673 | M |
| USNM | 96821 | 1899 | MEX | 23.269672 | -106.433106 | F |
| USNM | 98077 | 1898 | MEX | 22.835248 | -105.787437 | F |
| USNM | 33705 | 1892 | MEX | 29.079003 | -110.946764 | F |
| USNM | 100509 | 1900 | MEX | 17.55885 | -92.943884 | F |
| USNM | 100511 | 1900 | MEX | 17.55885 | -92.943884 | M |
| USNM | 92963 | 1898 | MEX | 22.400273 | -97.935081 | M |
| USNM | 92964 | 1898 | MEX | 22.400273 | -97.935081 | F |
| USNM | 94092 | 1898 | MEX | 22.400273 | -97.935081 | M |
| USNM | 7846 | N/A | MEX | 18.855177 | -97.091325 | M |
| USNM | 54989 | 1893 | MEX | 19.417195 | -97.007315 | M |
| USNM | 65956 | 1894 | MEX | 18.428974 | -95.117066 | M |
| USNM | 78123 | 1896 | MEX | 17.984231 | -94.550149 | M |
| USNM | 90988 | 1897 | MEX | 22.642443 | -104.102195 | M |
| USNM | 332425 | 1962 | NIC | 12.276025 | -86.36593 | M |
| USNM | 332426 | 1962 | NIC | 12.276025 | -86.36593 | M |
| USNM | 332429 | 1962 | NIC | 12.276025 | -86.36593 | M |
| USNM | 332430 | 1962 | NIC | 12.276025 | -86.36593 | F |
| USNM | 332431 | 1962 | NIC | 12.276025 | -86.36593 | M |
| USNM | 332432 | 1962 | NIC | 12.276025 | -86.36593 | F |
| USNM | 332433 | 1962 | NIC | 12.276025 | -86.36593 | M |
| USNM | 332434 | 1962 | NIC | 12.276025 | -86.36593 | F |
| USNM | 334580 | 1962 | NIC | 12.276025 | -86.36593 | M |
| USNM | 334581 | 1962 | NIC | 12.276025 | -86.36593 | F |
| USNM | 334582 | 1962 | NIC | 12.41721 | -86.645137 | F |
| USNM | 337522 | 1963 | NIC | 12.550278 | -87.166108 | F |
| USNM | 337525 | 1963 | NIC | 12.550278 | -87.166108 | F |
| USNM | 337526 | 1963 | NIC | 12.550278 | -87.166108 | F |
| USNM | 337531 | 1963 | NIC | 13.018815 | -85.835485 | F |
| USNM | 337653 | 1962 | NIC | 12.41721 | -86.645137 | F |
| USNM | 337654 | 1962 | NIC | 12.41721 | -86.645137 | M |
| USNM | 337841 | 1963 | NIC | 11.324662 | -85.70739 | F |
| USNM | 337842 | 1963 | NIC | 11.324662 | -85.70739 | M |
| USNM | 337843 | 1963 | NIC | 11.324662 | -85.70739 | M |
| USNM | 337846 | 1963 | NIC | 11.324662 | -85.70739 | F |
| USNM | 337847 | 1963 | NIC | 11.324662 | -85.70739 | F |
| USNM | 11422 | 1865 | MEX | 20.974654 | -89.613131 | F |
| USNM | 11425 | 1865 | MEX | 20.974654 | -89.613131 | F |
| USNM | 108495 | 1901 | MEX | 20.504898 | -86.925979 | F |
| USNM | 108496 | 1901 | MEX | 20.504898 | -86.925979 | F |
| USNM | 108497 | 1901 | MEX | 20.504898 | -86.925979 | F |
| USNM | 108499 | 1901 | MEX | 20.504898 | -86.925979 | M |
| USNM | 172068 | 1902 | MEX | 20.933487 | -89.016588 | M |
| USNM | 100531 | 1900 | MEX | 19.843214 | -90.50253 | M |
| USNM | 18342 | 1890 | USA | 29.36306 | -100.929094 | M |
| USNM | 24359 | 1890 | USA | 29.242485 | -100.790865 | M |
| USNM | 14909 | 1877 | USA | 25.900031 | -97.472453 | F |
| USNM | 31909 | 1891 | USA | 27.836771 | -97.492465 | M |
| USNM | 31415 | 1891 | USA | 27.7917 | -97.395474 | M |
| USNM | 32691 | 1892 | USA | 25.900031 | -97.472453 | F |
| USNM | 33131 | 1892 | USA | 25.900031 | -97.472453 | F |
| USNM | 33132 | 1892 | USA | 25.900031 | -97.472453 | M |
| USNM | 63130 | 1893 | USA | 29.301431 | -100.41757 | F |
| USNM | 63131 | 1893 | USA | 29.301431 | -100.41757 | M |
| USNM | 143137 | 1893 | USA | 29.301431 | -100.41757 | M |
| USNM | 61841 | 1895 | USA | 27.35493 | -81.043848 | M |
| USNM | 61843 | 1895 | USA | 27.324717 | -81.034807 | M |
| USNM | 61844 | 1895 | USA | 27.324717 | -81.034807 | M |
| USNM | 63997 | 1894 | USA | 27.433725 | -81.034504 | M |
| USNM | 63998 | 1894 | USA | 27.388499 | -81.037855 | F |
| USNM | 71753 | 1895 | USA | 24.567871 | -81.761412 | F |
| USNM | 79113 | 1895 | USA | 28.793453 | -81.027767 | M |
| USNM | 79116 | 1896 | USA | 28.793453 | -81.027767 | M |
| USNM | 111239 | 1902 | USA | 27.966209 | -81.451962 | M |
| USNM | 111262 | 1902 | USA | 27.966209 | -81.451962 | F |
| USNM | 263699 | 1938 | USA | 29.155318 | -81.861353 | F |
| USNM | 348115 | 1970 | USA | 30.907265 | -86.516541 | M |
| USNM | 111274 | 1902 | USA | 27.966209 | -81.451962 | M |
| USNM | 231584 | 1919 | USA | 25.493869 | -80.482784 | M |
| USNM | 33060 | 1892 | USA | 31.724426 | -81.474712 | F |
| USNM | 223934 | 1917 | USA | 30.927461 | -82.295943 | M |
| USNM | 34290 | 1892 | USA | 30.261134 | -91.990976 | F |
| USNM | 139776 | 1905 | USA | 29.702158 | -92.211705 | M |
| USNM | 247171 | 1926 | USA | 29.706483 | -91.195489 | M |
| USNM | 33126 | 1892 | USA | 30.311638 | -89.339036 | M |
| USNM | 33849 | 1892 | USA | 30.311638 | -89.339036 | F |
| USNM | 186544 | 1885 | USA | 32.433683 | -80.674068 | F |
| USNM | 32431 | 1892 | USA | 28.703537 | -95.962361 | F |
| USNM | 32433 | 1892 | USA | 28.904226 | -96.183146 | F |
| USNM | 203535 | 1914 | USA | 34.851795 | -87.527773 | F |
| USNM | 207189 | 1915 | USA | 33.995097 | -87.099042 | M |
| USNM | 197061 | 1900 | USA | 34.496837 | -91.561318 | M |
| USNM | 347670 | 1967 | USA | 38.362158 | -87.710899 | M |
| USNM | 347672 | 1962 | USA | 40.290583 | -85.423302 | M |
| USNM | 347673 | 1962 | USA | 40.429833 | -86.953337 | F |
| USNM | 506572 | 1968 | USA | 40.746064 | -86.170982 | M |
| USNM | 506573 | 1968 | USA | 40.840152 | -85.731262 | M |
| USNM | 268016 | 1938 | USA | 37.680097 | -84.41814 | F |
| USNM | 116197 | 1902 | USA | 38.977128 | -77.157349 | M |
| USNM | 397176 | 1970 | USA | 38.06781 | -75.567968 | M |
| USNM | 397187 | 1970 | USA | 38.06781 | -75.567968 | M |
| USNM | 235291 | 1920 | USA | 36.507346 | -92.244956 | M |
| USNM | 34867 | 1892 | USA | 36.668084 | -93.334739 | F |
| USNM | 360907 | 1963 | USA | 35.371924 | -77.96403 | M |
| USNM | 252999 | 1928 | USA | 39.290862 | -81.976513 | F |
| USNM | 36179 | 1892 | USA | 34.847296 | -95.566814 | F |
| USNM | 132448 | 1904 | USA | 34.151706 | -98.278902 | M |
| USNM | 132449 | 1904 | USA | 34.151706 | -98.278902 | M |
| USNM | 132450 | 1904 | USA | 34.151706 | -98.278902 | M |
| USNM | 132452 | 1904 | USA | 34.151706 | -98.278902 | M |
| USNM | 132453 | 1904 | USA | 34.151706 | -98.278902 | F |
| USNM | 132455 | 1904 | USA | 34.767786 | -98.592081 | M |
| USNM | 273946 | 1942 | USA | 34.688344 | -98.449463 | F |
| USNM | 273966 | 1938 | USA | 34.722082 | -98.700329 | F |
| USNM | 173088 | 1911 | USA | 32.785077 | -79.861392 | M |
| USNM | 34864 | 1892 | USA | 36.237234 | -88.087727 | F |
| USNM | 267411 | 1937 | USA | 36.356057 | -83.415341 | M |
| USNM | 267413 | 1937 | USA | 36.538467 | -87.360104 | M |
| USNM | 112025 | 1900 | USA | 29.953021 | -98.803901 | F |
| USNM | 186548 | 1886 | USA | 30.752681 | -99.234574 | M |
| USNM | 186547 | 1885 | USA | 30.752681 | -99.234574 | F |
| USNM | 348326 | 1972 | USA | 33.962807 | -98.775435 | F |
| USNM | 251440 | 1934 | USA | 38.091101 | -75.20495 | M |
| USNM | 19153 | 1890 | USA | 38.804835 | -77.046921 | M |
| USNM | 293195 | 1944 | USA | 37.228202 | -80.390484 | M |
| USNM | 349915 | 1971 | USA | 38.707186 | -77.156004 | M |
| USNM | 567500 | 1936 | USA | 37.228202 | -80.390484 | M |
| USNM | 589209 | 1972 | USA | 37.392989 | -80.559771 | F |
| USNM | 260240 | 1936 | USA | 38.429163 | -82.336847 | F |
| USNM | 396183 | N/A | USA | 39.056714 | -78.960104 | M |
| USNM | 600217 | 2010 | USA | 38.947802 | -77.49075 | M |
