# Supplementary material for: Playing by the rules? Phenotypic adaptation to temperate environments in an American marsupial
Source: PeerJ. 2018 Mar 27;6:e4512. doi: 10.7717/peerj.4512 (PMC5877449; doi:10.7717/peerj.4512)
Supplement: Table S3 — The temperature and precipitation variables are derived from the monthly temperature and rainfall values. The variables represent annual trends, seasonality and extreme or limiting environmental factors. [file peerj-06-4512-s005.docx]

**Table S3.** List of the bioclimatic and geographic variables measured in our study. The temperature and precipitation variables are derived from the monthly temperature and rainfall values. The variables represent annual trends, seasonality and extreme or limiting environmental factors.

| Bio1 = Annual mean temperature |
| --- |
| Bio2 = Mean diurnal range (mean of monthly (max temp - min temp)) |
| Bio4 = Temperature seasonality (standard deviation *100) |
| Bio10 = Mean temperature of warmest quarter |
| Bio11 = Mean temperature of coldest quarter |
| Bio12 = Annual precipitation |
| Bio15 = Precipitation seasonality (coefficient of variation) |
| Bio19 = Precipitation of coldest quarter |
| NDVIMAX = Normalized difference vegetation index maximum value |
| TREECOV = Vegetation continuous field product (percent tree coverage) |
| ELEV = Elevation |
| ROUGH = Surface moisture |
| LAT = Latitude |
| LONG = Longitude |
