# Supplementary material for: Playing by the rules? Phenotypic adaptation to temperate environments in an American marsupial
Source: PeerJ. 2018 Mar 27;6:e4512. doi: 10.7717/peerj.4512 (PMC5877449; doi:10.7717/peerj.4512)
Supplement: Table S4 — P-values for the spatial autocorrelation test are shown for each trait. All traits have significant values of spatial autocorrelation. [file peerj-06-4512-s006.docx]

**Table S4.** Moran’s I spatial autocorrelation test results for all phenotypic traits. P-values for the spatial autocorrelation test are shown for each trait. All traits have significant values of spatial autocorrelation.

| Trait | Moran’s I | *P*-value |
| --- | --- | --- |
| Body length | 0.0778 | < 0.0001 |
| Hindfoot length | 0.1090 | < 0.0001 |
| Tail length | 0.3106 | < 0.0001 |
| Ear length | 0.0956 | 0.0029 |
| Tail pigmentation | 0.4077 | < 0.0001 |
| Ear pigmentation | 0.4250 | < 0.0001 |
| Toe ventral pigmentation | 0.3918 | < 0.0001 |
| Toe dorsal pigmentation | 0.1927 | < 0.0001 |
| Rostrum lightness | 0.4667 | < 0.0001 |
| Temporal region lightness | 0.4870 | < 0.0001 |
| Cheek lightness |  |  |
| F | 0.1067 | 0.0002 |
| M | 0.1948 | < 0.0001 |
| Torso lightness |  |  |
| F | 0.1356 | < 0.0001 |
| M | 0.1033 | 0.0002 |

F: Females; M: Males.
