# Supplementary material for: Playing by the rules? Phenotypic adaptation to temperate environments in an American marsupial
Source: PeerJ. 2018 Mar 27;6:e4512. doi: 10.7717/peerj.4512 (PMC5877449; doi:10.7717/peerj.4512)
Supplement: Table S5 — P-values for the Moran’s I spatial autocorrelation test are shown for each trait. All models have negative spatial autocorrelation values, most of them are small and slightly significant or not significant. [file peerj-06-4512-s007.docx]

**Table S5.** Moran’s I spatial autocorrelation test for the residuals of the combined random forest models. P-values for the Moran’s I spatial autocorrelation test are shown for each trait. All models have negative spatial autocorrelation values, most of them are small and slightly significant or not significant.

| Traits | Best models including environmental and geographic variables | Moran’s I | *P*-value |
| --- | --- | --- | --- |
| Body Dimensions |  |  |  |
| Body length | LAT, Bio4, Bio11, Bio2, LONG | -0.1024 | 2.8 x 10^-06^ |
| Hindfoot length | Bio11, LONG, LAT, Bio4 | -0.0649 | 0.004 |
| Tail length | LAT, LONG, Bio4, Bio1, Bio11 | -0.0455 | 0.051 |
| Ear length | Bio1, LAT, Bio4, ROUGH, LONG | -0.0536 | 0.184 |
| Skin Pigmentation |  |  |  |
| Tail pigmentation | LONG, LAT, Bio4, Bio11, Bio15 | -0.0742 | 0.001 |
| Ear pigmentation | LONG, Bio15, Bio4, LAT | -0.0412 | 0.110 |
| Toe ventral pigmentation | LAT, Bio 19, LONG, Bio4, Bio11 | -0.0478 | 0.049 |
| Toe dorsal pigmentation | LAT, Bio11, Bio15, Bio1, LONG | -0.0475 | 0.046 |
| Face coloration |  |  |  |
| Rostrum lightness | LAT, Bio4, LONG, Bio11, Bio19 | -0.0520 | 0.031 |
| Temporal lightness | LONG, LAT, Bio4, Bio11, Bio15 | -0.0574 | 0.013 |
| Cheek lightness |  |  |  |
| F | LAT, Bio4, Bio11, Bio19, LONG | -0.0718 | 0.045 |
| M | LAT, Bio11, Bio4, Bio15, LONG | -0.0958 | 0.003 |
| Torso lightness |  |  |  |
| F | Bio4, Bio12, LAT, Bio11, LONG | -0.0403 | 0.292 |
| M | Bio4, Bio11, LAT, Bio12, LONG | -0.1487 | 1.9 x10^-06^ |

F: Females; M: Males.
